# Evaluation of the safety, pharmacokinetics, pharmacodynamics, and drug-drug interaction potential of a selective Lp-PLA2 inhibitor (GSK2647544) in healthy volunteers 

**DOI:** 10.5414/CP202565

**Published:** 2016-10-10

**Authors:** Kai Wu, Jianfeng Xu, Regan Fong, Xiaozhou Yao, Yanmei Xu, William Guiney, Frank Gray, Andrew Lockhart

**Affiliations:** 1 GlaxoSmithKline R&D, Shanghai, China,; 2 GlaxoSmithKline R&D, Philadelphia, PA,; 3 GlaxoSmithKline R&D, Research Triangle Park, NC, USA,; 4 GlaxoSmithKline R&D, Ware,; 5 GlaxoSmithKline R&D, Stevenage, and; 6 GlaxoSmithKline R&D, Cambridge, UK; Present addresses: Kai Wu: Wuxi Clinical Development Service, Shanghai, China; Regan Fong: UCB Biosciences Inc., RTP, USA; Xiaozhou Yao: Alexion Pharmaceuticals, Cambridge, USA; Frank Gray: Indivior UK Ltd., Slough, UK

**Keywords:** GSK2647544, lipoprotein-associated phospholipase A2, pharmacokinetics, pharmacodynamics, safety, phase 1

## Abstract

Abstract. Objective: To evaluate in healthy volunteers the safety, pharmacokinetics (PK), pharmacodynamics (PD), and drug-drug interaction (DDI) potential of GSK2647544, (a selective lipoprotein-associated phospholipase A2 (Lp-PLA2) inhibitor). Methods: Study 1 was a single-blind, randomized, placebo-controlled, crossover study with healthy male volunteers randomized to receive single escalating oral doses (0.5 – 750 mg) of GSK2647544. Study 2 was a single-blind, randomized, placebo-controlled study with healthy volunteers randomized to receive repeat doses (80 mg) of GSK2647544. The drug-drug interaction of GSK2647544 with simvastatin was also evaluated in study 2. Results: Across both studies GSK2647544 doses were generally well tolerated with no GSK2647544-related clinically significant findings. GSK2647544 was readily absorbed and its plasma concentration declined bi-exponentially with a terminal half-life ranging from 8 to 16 hours. Plasma exposure of GSK2647544 increased approximately dose-proportionally. There was GSK2647544 dose-dependent inhibition of plasma Lp-PLA2 activity, with a trough inhibition (12 hours after dose) of 85.6% after 7-day twice daily dosing. The administration of simvastatin concomitantly with GSK2647544 increased the overall exposure (area under the plasma concentration-time curve and maximum plasma concentration) of simvastatin and simvastatin acid by 3.6- to 4.3-fold and 1.5- to 3.1-fold, respectively. Conclusions: GSK2647544 was generally well tolerated and had a reasonable PK-PD profile. The clinically significant drug-drug interaction led to an early termination of study 2.

## Introduction 

Alzheimer’s disease (AD) is a progressive neurodegenerative disorder that accounts for 60 – 70% of > 47.5 million people affected worldwide by dementia [1]. The disease is characterized by cognitive deficits and over time affected individuals become profoundly cognitively and functionally impaired. AD remains an area of high unmet medical need as currently available treatment options for AD are limited and provide only modest, short-term symptomatic benefit [2]. 

Lipoprotein-associated phospholipase (Lp-PLA2), also known as platelet-activating factor acetylhydrolyse (PAF-AH), is a calcium-independent phospholipase A2 with proinflammatory activities that is primarily secreted by monocyte-derived macrophages. The enzyme circulates in plasma as a complex with low-density lipoprotein and, to a lesser extent, with high-density lipoprotein [3]. Lp-PLA2 has been studied extensively as a marker of cardiovascular risk, and inhibitors of the enzyme were developed to counter the atherogenic properties of oxidized low-density lipoproteins by inhibiting the production of proinflammatory and proapoptotic lipid mediators, lyso-phosphatidylcholine and non-esterified fatty acids. These proinflammatory lipids are thought to play an important role in driving the vascular inflammation [3]. 

Preliminary clinical evidence that targeting Lp-PLA2 may provide a novel treatment to slow the progression of AD comes from a phase 2a study with a non-central nervous system (CNS)-penetrant Lp-PLA2 inhibitor rilapladib, which demonstrated improved cognitive outcomes and changes to a number of mechanism- and disease-related biomarkers [4]. This study was based on findings from a diabetic mellitus (DM) and hypercholesterolemic (HC) pig model, in which treatment with darapladib (another Lp-PLA2 inhibitor) numerically reduced the extent of immunoglobulin-G brain parenchyma penetration suggesting a reduction in blood brain barrier leakage, and significantly lowered the total amount of brain amyloid-β peptide 1-42 deposition compared with untreated DM/HC pigs [5]. 

Evidence indicates that Lp-PLA2 is also present in the human central nervous system (CNS) [4] and that its specific activity can be detected in cerebrospinal fluid [5, 6]. Immunohistochemical evidence indicates that its expression is primarily associated with microglia [GSK data on file]. Pharmacological inhibition of this central pool of Lp-PLA2 has the potential to provide an additional interventional mechanism for the treatment of AD and other neurological indications. GSK2647544 is an orally available, selective inhibitor of Lp-PLA2 [data on file], and data from a human PET biodistribution study indicates that it is a brain penetrant in humans with a mean whole brain volume of distribution (V_T_) of 0.56 (Clintrials.gov: NCT01924858; http://www.gsk-clinicalstudyregister.com/study/117155#rs). 

The aims of the present clinical studies were to assess (1) the overall safety, tolerability, pharmacokinetic (PK), and pharmacodynamic (PD) profiles of GSK2647544 in healthy subjects and (2) the potential for an in-vivo drug-drug interaction (DDI) due to its potent inhibition of cytochrome P450 3A4 in pooled human liver microsomes, wherein IC50 values of 0.12 µM (probe substrate atorvastatin) and 2.9 µM (probe substrates midazolam and nifedipine) were observed. Study 1 was the first-in-human (FIH) study that used single escalating oral doses of GSK2647544 (0.5 – 750 mg) and had primary outcomes on safety and tolerability and secondary outcomes relating to plasma PK and PD. Although study 2 was designed with the overall intent to provide safety and PK data that would support patient studies in AD, the first part of the study was designed to address the potential cytochrome P450 3A4 (CYP3A4) DDI liability noted above, as this had the potential to negatively impact on the overall developability of the molecule. As described in the current paper, GSK2647544 was found to be a moderate to strong CYP3A4 inhibitor in the first cohort of subjects; this led to early termination of study 2. 

## Methods 

Study 1 (GSK protocol number LPC116698; Clintrials.gov NCT01702467) was conducted at GlaxoSmithKline Medicines Research Unit, Prince of Whales Hospital, Randwick, Australia. Study 2 (GSK protocol number 200592; Clintrials.gov NCT01978327) was conducted at Parexel Early Phase Unit, Northwick Park Hospital, Harlow, United Kingdom. Both studies were conducted according to the principles of the Declaration of Helsinki and Good Clinical Practice guidelines, and approved by the institutional review boards of participating facilities, the Bellberry Human Research Ethics Committees in Australia (study 1) and the National Research Ethics Service in London (study 2). All subjects gave their written informed consent. The same manufacturing batch of GSK2647544 was used in both clinical studies. 

### Study design 

**Study 1: single ascending dose FIH study**

This single-blind, randomized, placebo-controlled, crossover study was designed to assess PK, PD, safety, and tolerability of single doses of GSK2647544. Three cohorts consisting of 24 healthy male volunteers (8 each cohort, 3 : 1 ratio of active : placebo) were randomized to receive single escalating oral doses of GSK2647544 or placebo (all doses were provided fasted unless stated): cohort 1 (0.5, 2, 8, and 40 mg of GSK2647544 and placebo fasted), cohort 2 (8, 60, and 125 (fed) mg of GSK2647544 and placebo), and cohort 3 (250, 325 (fed), 600 (fed), and 750 (fed) mg of GSK2647544 and placebo). The allometric scaling based on rat, dog, and monkey PK data predicted a 686-fold safety margin for AUC_(0-24)_ and a 1,882-fold safety margin for C_max_ of 0.5 mg, respectively, relative to the mean exposure observed at the no observed adverse effects level in animals. 

Subjects were screened for eligibility within 30 days prior to day 1, examined on day –1 for baseline physical and laboratory assessments, and monitored for safety and tolerability endpoints after dosing at protocol defined intervals until the end of follow-up. Subjects were admitted and stayed in the study center until all assessments were completed for each dosing session. Safety assessments included physical examinations, vital signs, clinical laboratory parameters (including blood biochemistry, hematology, and urinalysis), electrocardiography (ECG), and Columbia suicide-severity rating scale (CSSRS). 

Study drug was administered in capsules after a standard meal or after an overnight fast of at least 8 hours for the fasted condition. Dose escalations were initiated only after completion of the interim assessment and review of safety data from all subjects receiving GSK2647544 or placebo from the preceding cohort. Interim PK assessments were conducted to ensure maintenance of the safety margin. There was a minimum 7-day washout between dosing sessions and a follow-up 7 – 14 days after last dose. 

**Study 2: repeat ascending dose and DDI study**

**Figure 1.**
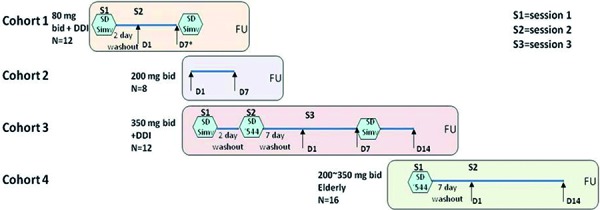
Schematic showing the overall design of study 2 with the four dosing cohorts shown, which should be read in conjunction with the study design summary provided in the Methods section. The study was terminated at the end of cohort 1 based on the findings of a moderate to strong CYP3A4 inhibition. ‘544 = GSK2647544; SD = single dose; Simv = simvastatin (10 mg); FU = follow-up; DDI = drug-drug interaction evaluation.

This single-blind, randomized, placebo-controlled study was designed to assess PK, PD, safety, and tolerability of repeat doses of GSK2647544. The study was originally planned to have four cohorts (Figure 1). The study was terminated at the end of cohort 1 based on interim analysis results which indicated a moderate to strong CYP3A4 inhibition. In cohort 1, 12 subjects were randomized to active (GSK2647544) or placebo (3 : 1 ratio). All the subjects went through 2 treatment sessions separated by a minimum 2-day washout period and a follow-up visit 7 – 14 days post last dose. Subjects were admitted and stayed in the study center until all assessments were completed for each dosing session. In session 1, the subjects received a single dose of 10 mg simvastatin in the morning. In session 2, the subjects received repeat doses of 80 mg b.i.d. or placebo for 7 days and a single dose of 10 mg simvastatin on the morning of day 7. 

Screening and safety assessments were as described for study 1. Study drug was administered after a standard meal. The simvastatin clinical dose range is 5 – 80 mg once daily; to ensure an adequate safety margin, simvastatin 10 mg was used in the study. 

### Study population 

**Study 1**

Eligible subjects were between the ages of 18 and 55 years, with body weights > 50 kg and BMIs of 19 – 30 kg/m^2^, who were deemed to be in good health based on their medical history, a physical examination, and routine laboratory tests. Subjects were excluded if they had history or existing conditions of asthma, anaphylaxis, anaphalactoid reactions, severe allergic responses, hypercoagulable state, thrombosis, biliary tract disease, or liver disease. 

**Study 2**

Eligible subjects were between the ages of 18 and 64 years, with body weights > 50 kg and BMIs of 19 – 32 kg/m^2^, who were deemed to be in good health based on their medical history, a physical examination, and routine laboratory tests. Subjects were excluded if they had history or existing conditions of asthma, anaphylaxis, anaphalactoid reactions, severe allergic responses, hypercoagulable state, thrombosis, biliary tract disease, or liver disease. 

### Pharmacokinetic and pharmacodynamic sampling 

**Study 1**

Separate blood samples for determination of GSK2647544 plasma concentrations and plasma Lp-PLA2 enzyme activity were collected at predose, 0.5, 1, 2, 4, 6, 8, 12, 24, 36, 48, and 72 hours post dose. 

**Study 2**

Blood samples for determination of GSK2647544 plasma concentrations were collected at predose in the morning, 0.5, 1, 2, 4, 6, 8, and 12 hours post morning dose on day 1 and 7, predose in the morning on day 3, 4, and 5, and at 36, 48, 60, and 84 hours post morning dose on day 7. Blood samples for determination of simvastatin and simvastatin acid plasma concentrations were collected at predose in the morning, 0.5, 1, 2, 4, 6, 8, 12, and 24 hours post morning dose. Blood samples for determination of Lp-PLA2 enzyme activity were collected at predose, 0.5, 1, 2, 4, 6, 8, and 12 hours post morning dose on day 1 and 7, and at 36, 48, 60, 84, and 120 hours post morning dose on day 7. 

### Bioanalytical methods 

GSK2647544 was extracted from human plasma by protein precipitation using 90/10 v/v acetonitrile/water with 0.1% ammonium solution containing an isotopically labeled internal standard ([^13^C_6_] GSK2647544). Extracts were analyzed by HPLC MS/MS using a TurboIonspray™ interface (column: C18, 50 × 2.1mm i.d. column type 1.7 µm, Waters, Elstree, UK) with positive ion multiple reaction monitoring. This method was validated over the range 0.5 – 500 ng/mL with a within-run precision of 1.2 – 8.1%, bias –11.1 – 7.2% and a between-run precision of 1.6 – 8.3%, bias –1.2 – 4.9%. Quality controls for run acceptance were prepared and analyzed with each batch of sample against separately prepared calibration standard to assess day-to-day performance of the assay. For the analysis to be acceptable, no more than 1/3 of the quality control results were to deviate from the nominal concentration by more than 15%, with at least 1 quality control result acceptable at each concentration. Quality control results from these studies met these acceptance criteria. 

Simvastatin and simvastatin acid were extracted from human plasma using liquid-liquid extraction (internal standards: [^2^H_3_]-simvastatin and [^2^H_3_]-simvastatin acid]. A 75-µL volume of 100 mM ammonium acetate, 0.75% acetic acid was added to a well plate and frozen. A 100-µL matrix aliquot was added and fortified with 20 µL of internal standard working solution. The plate was vortexed and centrifuged and then the sample was transferred via a Hamilton NIMBUS liquid handling system (Hamilton, Reno, NV, USA) to a 96-well plate Isolute SLE+ plate (Biotage, Charlotte, NC, USA) and absorbed into the plate via positive pressure. After 5 minutes, the sample was eluted with 800 µL of methyl-t-butyl ether and dried under nitrogen gas. The sample was reconstituted with 100 µL of acetonitrile, vortexed, then mixed with 100 µL of 1.0 mM ammonium acetate, 0.01% acetic acid. A 30-µL volume of final extract was injected and analyzed via HPLC with MS/MS detection m/z 441 to 325 simvastatin, and 435 to 319 simvastatin acid). This method was validated over the range 0.100 – 50.0 ng/mL with a within-run precision of –9.92 – 5.78%, bias 0.844 – 11.1% and a between-run precision of –2.65 – 0.0897%, bias 5.05 – 7.84% for simvastatin and with a within-run precision of –5.65 – 9.17%, bias 1.33 – 9.29% and a between-run precision of –0.961 – 3.81%, bias 4.29 – 6.47% for simvastatin acid. Quality controls for run acceptance were prepared and analyzed with each batch of sample against separately prepared calibration standard to assess day-to-day performance of the assay. For the analysis to be acceptable, no more than 1/3 of the quality control results were to deviate from the nominal concentration by more than 15%, with at least 1 quality control result acceptable at each concentration. Quality control results from this study met these acceptance criteria. 

### Lp-PLA2 enzyme activity assay 

Blood samples of 2 mL were drawn into K2-EDTA tubes, inverted 8 – 10 times to mix, and centrifuged at ~ 1,600 × g for 15 minutes at room temperature to separate the plasma from the cells. Plasma samples were separated and frozen at –20 °C or lower within 30 minutes of collection. Lp-PLA2 activity was measured with a colorimetric assay, in which a plasma sample reacted with a substrate, 1-myristoyl-2-(4-nitrophenylsuccinylphosphatidylcholine), in a well of a 96-well microtiter plate. The change in the absorption at 405 nm in kinetic mode was measured with a microtiter plate analyzer. 

### Pharmacokinetic analysis 

PK analyses of individual plasma GSK2647544, simvastatin, and simvastatin acid concentration-time profiles were conducted by non-compartment analysis using the Model 200 (for extravascular administration) of WinNonlin Professional (Pharsight Corporation, Mountain View, CA, USA). Actual elapsed time from dosing was used to estimate all individual plasma PK parameters. C_max_ and the first time to reach C_max_ (t_max_), were the actual observed values. The terminal plasma elimination rate constant (lz) was estimated from log linear regression analysis of the terminal phase of the plasma concentration-time profile. The associated apparent terminal elimination half-life (t_1/2_) was calculated as t_1/2_ = ln2/lz. The area under the plasma concentration-time curve from time zero extrapolated to infinity (AUC_(0–∞)_)was calculated by the linear up/logarithmic down trapezoidal method. The area under the plasma concentration-time curve from time zero to the last time of quantifiable concentration (AUC_(0–t)_) and from time zero to 12 hours post dose (AUC_(0–12)_) were also calculated. 

### Pharmacodynamic analysis 

The effects of GSK2647544 on plasma Lp-PLA2 activity were expressed as percent inhibition of plasma Lp-PLA2 activity relative to baseline, i.e., 100% × ((Baseline activity level – postdose activity level)/baseline activity level). Time course data for percent inhibition of plasma activity was descriptively and graphically summarized. 

### Population PK-PD analyses 

Population PK and PK-PD analyses of study 1 were performed using non-linear mixed effects modeling utilizing NONMEM^®^ version 7.2 (ICON Development Solutions, Ellicott City, MD, USA). The established models were then used to simulate Lp-PLA2 activity inhibition as a basis for selecting dose and dose regimen for subsequent clinical studies. 

A compartmental PK model was fitted to the PK data to characterize the dose-exposure relationship of GSK2647544. An inhibitory E_max_ PD model was fitted to the Lp-PLA2 activity data as in equation 1, where: Cp is the observed GSK2647544 plasma concentration at the same time of Lp-PLA2 activity sampling; E0 is the observed Lp-PLA2 baseline activity; β is a slope factor; IC50 is the GSK2647544 plasma concentration required to inhibit 50% of Lp-PLA2 activity from baseline. Once the PK and PK-PD models were established, the PK model was used to simulate steady-state trough concentrations of GSK2647544 and the PK-PD model was used to simulate Lp-PLA2 activity inhibition at these concentrations. Simulations were conducted for 1,000 subjects, who were randomly selected using the between-subject variability estimated from PK and PK-PD models, at a range of doses given once or twice daily. Dose response relationship for plasma Lp-PLA2 activity inhibition was then established based on the simulation results. 

Lp-PLA2 activity = E0 × (1 – Cp/(Cp + IC50)) Equation 1 

### Statistical analysis 

**Study 1**

A statistical analysis was performed to assess dose proportionality on the exposure of GSK2647544 following single dose administration. The analysis was performed on log_e_-transformed AUC_(0–∞)_, AUC_(0–t)_, and C_max_. For each of these parameters, a mixed-effect power model was fitted with log_e _(dose) as a fixed effect and individual subject intercept parameter fitted as random effects. The slope on log_e_ (dose) was estimated from the model and the corresponding 90% confidence interval was calculated. A slope estimate of 1 indicates a dose-proportional increase. 

**Study 2**

A statistical analysis was performed to separately assess the DDI effect on simvastatin and simvastatin acid by GSK2647544. Following log_e_-transformation, C_max_ and AUC_(0–∞)_ of simvastatin and simvastatin acid were each analyzed using a mixed effects model with fixed effect terms for treatment. Subjects were treated as a random effect in the model. Point estimates and their associated 90% confidence intervals were constructed for the differences between session 2 (simvastatin administered together with GSK2647544) and session 1 (simvastatin administered alone). These point estimates and their associated 2-sided 90% confidence intervals were then back-transformed to the point estimates and 2-sided 90% confidence intervals for the ratios between session 2 and 1. 

## Results 

### Subject disposition 

The subject disposition and demographics in all subjects of the two studies are summarized in Table 1. 

**Study 1**

27 male subjects with an average age of 32 years (range 18 – 52 years) were randomized. 24 of the subjects were White, 2 were of mixed race, and 1 was Asian. 4 of the randomized subjects were withdrawn, 2 for adverse events (AEs), 1 at the investigator’s discretion, and 1 for a personal reason. The safety, PK, PD, and PK-PD populations comprised all 27 subjects. 

**Study 2**

A total of 12 male subjects with an average age of 30 years (range 19 – 54 years) were randomized in cohort 1. 10 of the subjects were White, 1 was African, and 1 was Asian. All the randomized subjects completed the study. The safety, PK, and PD populations comprised all 12 subjects. 

### Safety and tolerability 

The incidence of AEs reported in the two studies and the most frequent AEs, which were reported by at least 1subject on active treatment in at least 1 study, are summarized in Tables 2 and Table 3. 

**Study 1**

22 subjects (81%) reported at least 1 AE during the study. The most commonly reported AEs (≥ 2 incidences) were headache (11 subjects (41%)), contact dermatitis (9 subjects (33%)), myalgia (4 subjects (15%)), presyncope (4 subjects (15%)), ventricular tachycardia (3 subjects (11%)), dizziness (2 subjects (7%)), fatigue (2 subjects (7%)), nasal congestion (2 subjects (7%)), and nasopharyngitis (2 subjects (7%)). All AEs were mild or moderate in intensity except 1 AE of severe nausea reported by a subject after placebo was administered. No apparent dose-dependent increase in frequency and severity of AEs was observed. Three subjects were noted on telemetry to have non-sustained ventricular tachycardia lasting between 3 and 5 beats; 2 (7%) subjects were withdrawn from the study. 

A 27-year-old male subject had a non-sustained, 5-beat run of ventricular tachycardia noted on telemetry ECG ~ 30 hours after dosing with 0.5 mg of GSK2647544. Unscheduled biochemistry and troponins were normal, and there was no evidence of QT prolongation or any other morphological change on 12-lead ECG monitoring. This subject did not have any further episodes; however, as a precaution he was withdrawn from the study. In the investigator’s opinion this was unrelated to study drug. 

A 21-year-old male subject had brief palpitation with concurrent display of a short 3-beat run of ventricular tachycardia on telemetry ~ 36 hours after dosing with 8 mg of GSK2647544. Unscheduled biochemistry and troponins were normal, and there was no evidence of QT prolongation or any other morphological change on 12-lead ECG monitoring. In the investigator’s opinion this was unrelated to study drug. The subject continued and completed the study with no further occurrences in subsequent dosing sessions with 60 mg and 125 mg of GSK2647544. 

A 28-year-old male subject had a 4-beat, non-sustained run of ventricular tachycardia ~ 7.5 hours after dosing with 125 mg of GSK2647544. There was no evidence of QT prolongation, he was asymptomatic at the time, physical exam was normal and unscheduled biochemistry and troponin were normal. He later had a ventricular couplet at 11.5 and 37 hours postdose. As the event occurred a few hours after dosing, this event was considered possibly related to study drug by the investigator, and therefore the subject was withdrawn from the study. 

There were no clinically significant changes that were considered to be related to GSK2647544 in 12-lead ECG. There were no clinically significant vital sign or laboratory parameter changes reported. No subject had episodes of suicidal ideation or behavior during the study. There were no serious adverse events (SAEs) or deaths reported in this study. 

**Study 2**

9 subjects (75%) reported AEs during the study. The most commonly reported AEs (≥ 2 incidences) were headache, abdominal discomfort, and application site rash, each in 3 subjects (25%). All other AEs were single occurrence. All the reported AEs were mild in intensity and resolved without treatment. None of the AEs led to withdrawal from study. The most commonly reported GSK2647544-related AEs (≥ 2 incidences) were abdominal discomfort (3 subjects (33%)) and headache (2 subjects (22%)). No clinically significant findings of physical exams, vital signs, ECG, or clinical laboratory assessments were found following treatments with simvastatin, GSK2647544, or placebo. No subjects had ventricular tachycardia events. No subjects had suicidal ideation or behaviors in any treatment groups. No SAEs or deaths were reported. 

## Pharmacokinetics 

### GSK2647544 

**Study 1**

**Figure 2.**
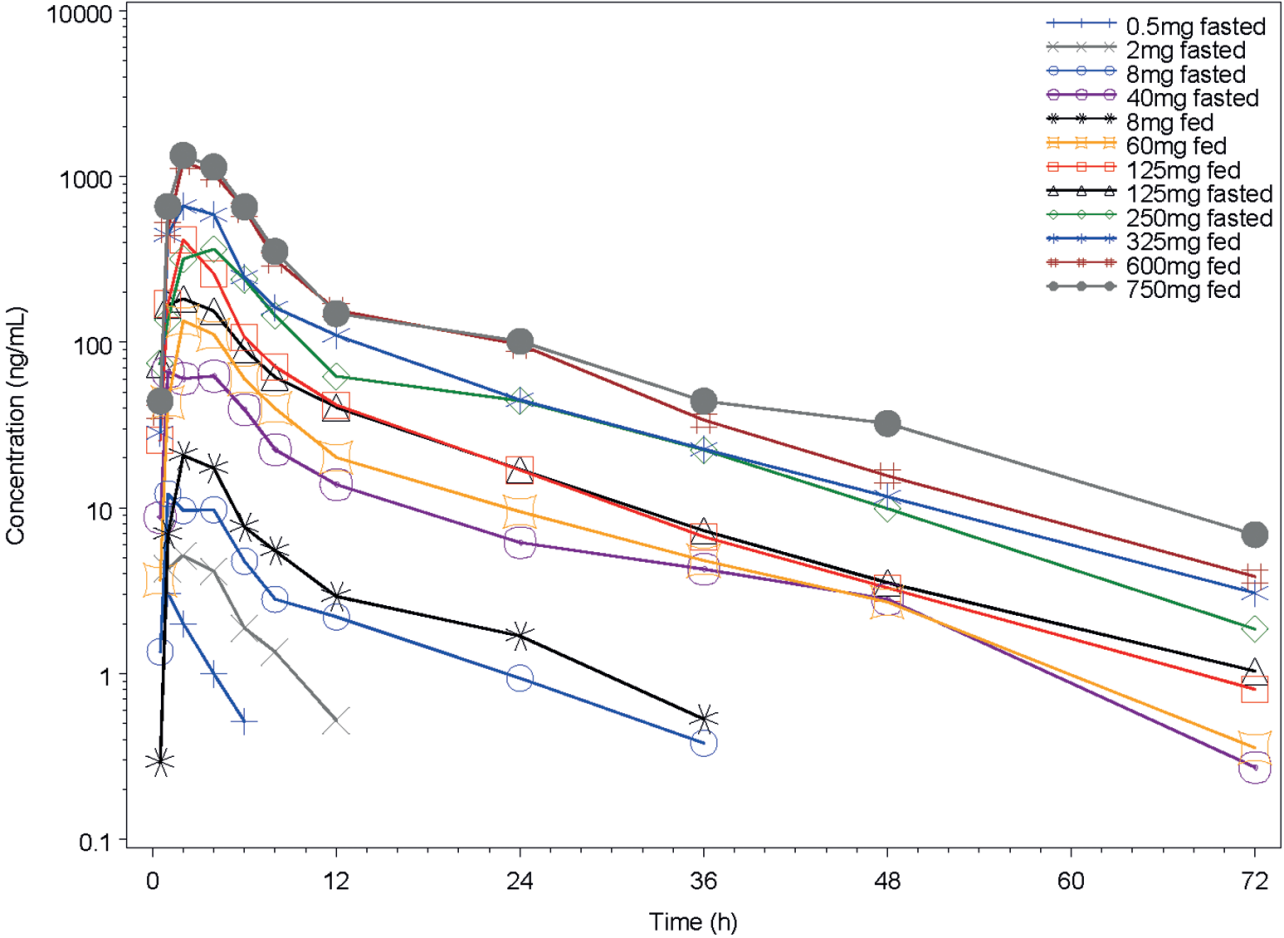
GSK2647544 median plasma concentration-time profiles following single doses of GSK2647544 0.5 – 750 mg.

GSK2647544 median plasma concentration-time profiles over 72 hours for single doses (0.5 – 750 mg) are shown in Figure 2. GSK2647544 PK parameters are summarized in Table 4. The median t_max_ ranged from 1 to 4 hours for all doses. Plasma concentrations of GSK2647544 declined bi-exponentially following the peak for doses ≥ 8 mg, with the median values above the lower limit of quantification (LLQ) up to 72 hours. The plasma concentration declined mono-exponentially following the peak for lower doses (0.5 and 2 mg), with the median values above the LLQ only up to 12 hours. The t_1/2_ of GSK2647544 was comparable for the doses 8 – 750 mg, ranging from 8 to 13 hours, in both fasted and fed states. The plasma exposure of GSK2647544 increased with increasing dose. C_max_, AUC_(0–t)_, and AUC_(0–∞)_ increased approximately dose-proportionally in the fasted (8 – 250 mg) and fed (8 – 325 mg) state. A comparison between fed and fasted state following 8 mg and 125 mg doses, showed that both overall and peak exposure to GSK2647544 was slightly higher (on average 1.3 to 2.2-fold) under fed conditions. 

**Study 2**

**Figure 3.**
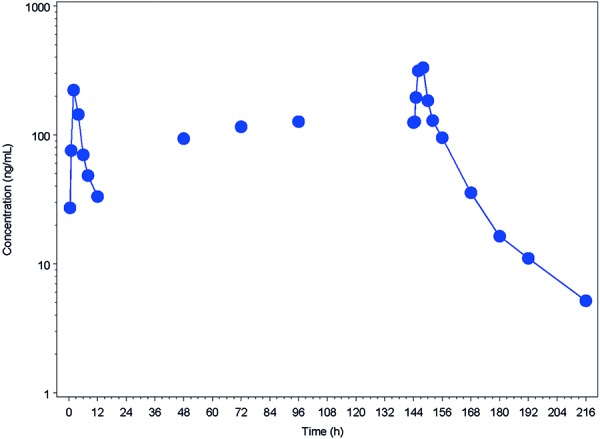
GSK2647544 median plasma concentration–time profiles following 7-day repeat b.i.d. dose of 80 mg GSK2647544.

GSK2647544 median plasma concentration-time profiles following 7-day repeat b.i.d. dose of 80 mg are shown in Figure 3. GSK2647544 PK parameters are summarized in Table 5. The median t_max_ on days 1 and 7 were 2 and 4 hours, respectively. The t_1/2_ following 7-day repeat dose was ~ 16 hours. There was 1.8-fold and 2.1-fold observed accumulation for C_max_ and AUC_(0–12)_, respectively, following 7-day repeat dose administration. Visual inspection of the mean trough plasma concentrations from day 3 to day 7 suggested the achievement of steady state by day 7. 

### Simvastatin and simvastatin acid 

**Figure 4.**
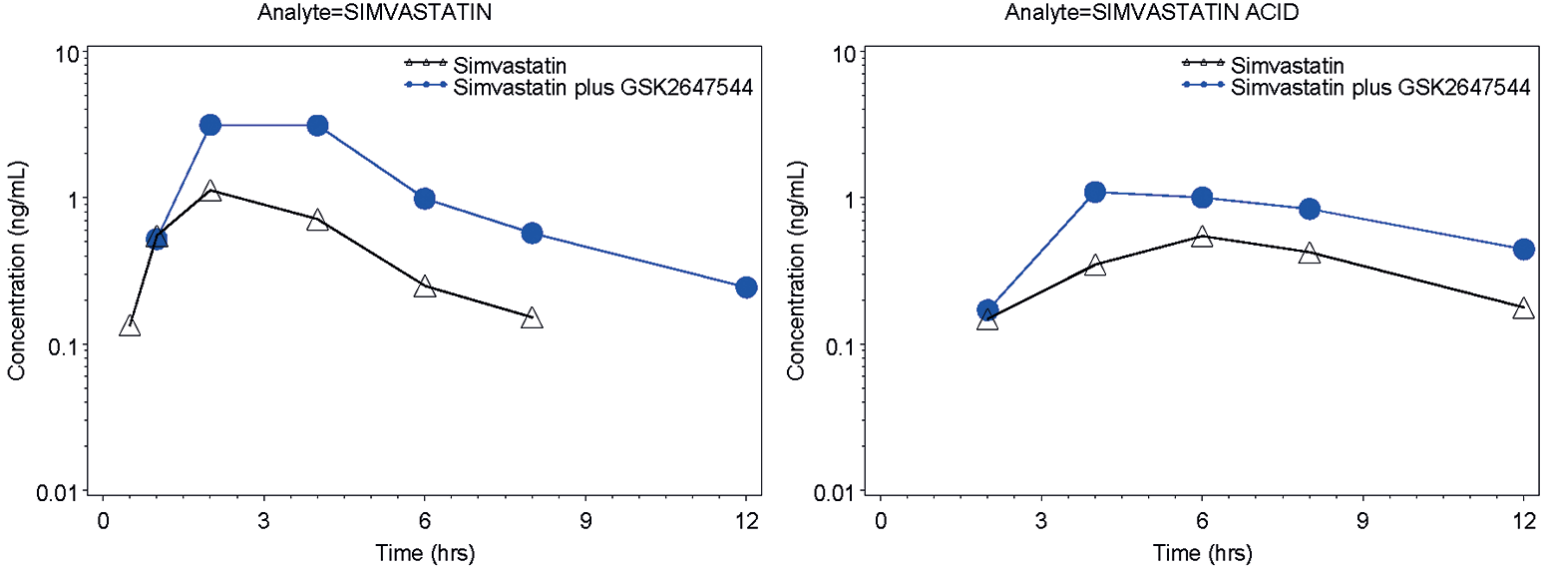
Median plasma concentration-time profiles of simvastatin and simvastatin acid following single dose of simvastatin, with and without GSK2647544.

Simvastatin and simvastatin acid median plasma concentration-time profiles and their PK parameters following single doses of simvastatin with and without GSK2647544 are provided in Figure 4 and Table 6. For simvastatin, the median t_max_ was 2 hours following administration of simvastatin alone and in combination with GSK2647544. For simvastatin acid, the median t_max_ values were 4 and 6 hours, following administration of simvastatin alone and in combination with GSK2647544, respectively. On average, for simvastatin and simvastatin acid, the exposure (AUC and C_max_) was 3.6- to 4.3-fold and 1.5- to 3.1-fold higher, respectively, following administration of simvastatin concomitantly with GSK2647544 compared to administration of simvastatin alone (Table 6). 3 of the 9 subjects had individual fold-changes in simvastatin AUC of > 5 (5.5, 8.9, and 9.5). The lowest fold-change in simvastatin AUC was 2.2. 

### Pharmacodynamics 

**Figure 5.**
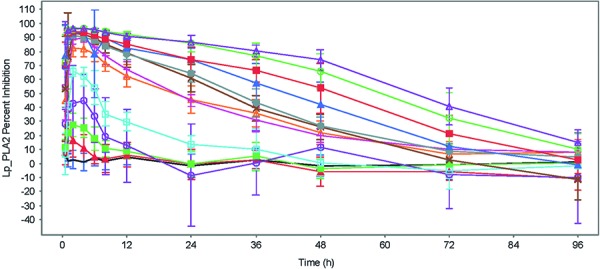
Mean (± SE) time course of % inhibition of plasma Lp-PLA_2_ enzyme activity following single doses of GSK2647544 0.5 – 750 mg.

The mean percent inhibition of plasma Lp-PLA2 activity from baseline following each single dose of GSK2647544 was plotted against observation time points (Figure 5). Inhibition was generally dose-dependent, peaking between 1 and 4 hours postdose and decreasing over time until back to predose baseline level or to the end of measurement period. 

**Figure 6.**
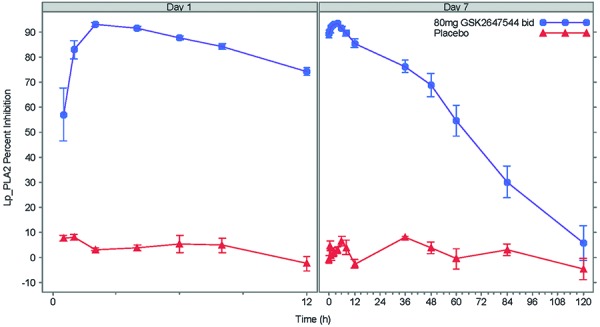
Mean (± SE) time course of % inhibition of plasma Lp-PLA_2_ enzyme activity following repeat b.i.d. doses of GSK2647544 80 mg.

The mean percent inhibition of plasma Lp-PLA2 activity from baseline following repeat b.i.d. dose of 80 mg GSK2647544 was plotted against observation time points (Figure 6). On day 7, peak inhibition was achieved at ~ 2 hours (median t_max_, 2 hours, range 1 – 4 hours). The mean trough inhibition (at 12 hours) from baseline was 85.6% (95% CI, 81.6 – 89.7%). 

### Population PK-PD 

**Figure 7.**
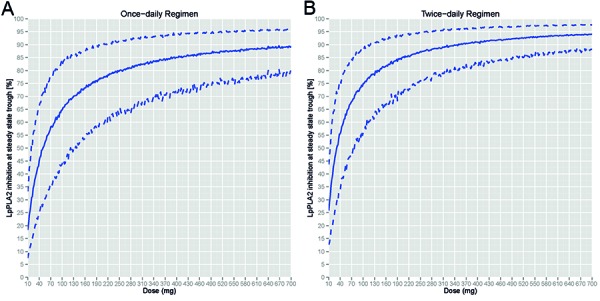
Predicted dose-response for trough Lp-PLA2 activity inhibition at steady state after once-daily or twice-daily repeat dosing of GSK2647544. Solid lines represent median response of 1,000 simulated subjects; dashed lines represent 5^th^ and 95^th^ percentile response of 1,000 simulated subjects.

PK and plasma Lp-PLA2 enzyme activity data from all 27 subjects of study 1 were included in the population PK and PK-PD analyses. A 2-compartment model with first-order absorption described the plasma concentration time course well, and PK parameters are summarized in Table 7. The direct inhibitory effect of PK on PD was well described by the E_max_ model (Equation 1) and estimated the IC50 to be 11.6 ng/mL. Based on the established models, dose-response curve for the Lp-PLA2 enzyme activity inhibition from baseline at the steady-state trough concentration was plotted (Figure 7). A lower daily dose was required for a twice-daily regimen than a once-daily regimen to reach the same level of PD response. For example, 142 mg daily dose and 250 mg daily dose were required to reach 80% Lp-PLA2 enzyme activity inhibition for a twice-daily and a once-daily regimen, respectively. 

## Discussion and conclusion 

GSK2647544 is a potent and specific Lp-PLA2 inhibitor that was under investigation for the treatment of AD. Results demonstrated that GSK2647544 was generally well tolerated by healthy subjects for single oral doses up to 750 mg and repeat 7-day b.i.d. oral dose of 80 mg. There were no SAEs and dose-limiting AEs observed in the study population. All GSK2647544-related AEs were mild or moderate in intensity, with headache being the most frequent AE in the single-dose study and abdominal discomfort being the most frequent AE in the repeat-dose study. There were no clinically significant findings of physical exams, vital signs, ECG, or clinical laboratory assessments. To our knowledge this is the first brain-penetrant Lp-PLA2 inhibitor that has been evaluated in clinical studies, and there was no evidence for increased incidence of CNS-related AEs. 

Episodes of asymptomatic cardiac arrhythmias, including unsustained ventricular tachycardia, are observed in healthy volunteers and the challenge in early clinical studies is to distinguish drug-induced effects from background incidence [8, 9, 10, 11]. A number of factors (lack of preclinical QT liability, lack of QT changes before or after arrhythmias, lack of dose or exposure response, and lack of recurrence on repeat dosing) make it unlikely that the unsustained ventricular tachycardias were related to GSK2647544 pharmacology or dosing. However, this is a specific focus of evaluation for NCEs, and ECG monitoring would be maintained in any future studies. 

After single-dose oral administration, GSK2647544 was readily absorbed with a t_max_ of 1 – 4 hours. GSK2647544 plasma concentration declined bi-exponentially for doses ≥ 8 mg and mono-exponentially for doses < 8 mg. The dose-dependency for the shape of plasma concentration time-course was probably due to the second phase of concentration time-course decline mostly being below the limit of quantification for doses < 8 mg. For the same reason, the terminal half-life was shorter for doses < 8 mg compared with higher doses. Comparison of PK of GSK2647544 in the fasted condition and in the fed condition demonstrated increased exposure and decreased between-subject variability. However, t_max_ was not affected by food conditions. The PK of GSK2647544 after repeat dose oral administration was consistent with the PK after single dose oral administration with t_max_ and t_1/2_ after repeat dose being similar to those after single dose. 

In-vitro studies indicated GSK2647544’s potential as a CYP3A4 inhibitor. Results from co-administration of GSK2647544 and simvastatin indicated that in-vitro potency against CYP3A4 has translated into a clinically significant effect. According to FDA guideline, GSK2647544 is defined as a moderate to strong inhibitor of CYP3A4 based on the observed 2.2- to 9.5-fold increase of AUC with the individual data [12]. 

AD is associated with progressive deficits in cognitive function and, due to age, subjects are typically on a wide range of medications to address other underlying conditions. Several classes of medications that are likely to be prescribed in AD patients [13] are also sensitive to inhibition of CYP3A4, including neuroleptics/antipsychotics, antiarrhythmics, antihypertensives, antidepressants, statins, and benzodiazepines. A number of drugs from these classes are contraindicated for use with CYP3A4 inhibitors (e.g., alprazolam, dofetilide, eplerenone, felodipine, nisoldipine, pimozide, quetiapine, simvastatin, thioridazine, triazolam, ziprasidone), and it would be anticipated that dose adjustment and/or additional safety monitoring would be required for other potential CYP3A4 substrates as part of a development plan. There is also the potential that despite these precautions clinically significant effects may only be unmasked at a late development stage or once the drug was on the market resulting in significant patient safety issues, delays to or additional regulatory filings, and/ or ultimately a withdrawal of the drug from the market. Although there is the potential to mitigate these risks through alterations to the dosing schedule of either GSK2647544 or potential “victim” drugs, the cognitive deficits and polypharmacy associated with AD were felt to effectively negate this approach. Considering the extent of the observed CYP3A4 inhibition and these additional factors, a decision was therefore taken to stop further dosing in study 2 and ultimately to terminate the study. 

There was a clear correlation between the plasma GSK2647544 concentration and inhibition of plasma Lp-PLA2 enzyme activity with no temporal dissociation after single dose of GSK2647544. The PK-PD modeling further characterized the relationship quantitatively with a direct-effect inhibitory E_max_ model. Based on the model prediction, twice-daily regimen was selected over once-daily regimen to be evaluated in study 2 because less total daily dose was required for twice-daily than once-daily regimen to reach the same level of Lp-PLA2 enzyme activity inhibition. In addition to the regimen selection, the model-based prediction also provided a useful guidance for the dose selection in study 2. The 80 mg b.i.d. was selected as the starting dose in study 2 because the model-based dose-response curve (Figure 7) showed that 80 mg b.i.d. (160 mg daily dose) of GSK2647544 would inhibit ~ 82% plasma Lp-PLA2 enzyme activity at trough, which was considered as necessary for GSK2647544, an enzyme inhibitor to produce clinical effect. This model-predicted inhibition was consistent with the observation (86% inhibition). 

In summary, GSK2647544 was generally well tolerated and had a reasonable PK-PD profile. The finding of a clinically significant DDI liability led to the early termination of study 2. 

## Acknowledgment 

The authors wish to thank Gang Xu for his contribution to the study. 

## Conflict of interest 

The studies were sponsored and funded by GlaxoSmithKline (GSK). JX, YX, WG, and AL are employees of GSK R&D. FG, KW, XY, and RF were employees of GSK R&D when the studies were conducted. KW, JX, RF, XY, YX, WG, FG, and AL own GSK stocks. 

**Table 1.** Subject disposition and demographic information.

Study	Study 1			Study 2
Cohort	Cohort 1^1^	Cohort 2^2^	Cohort 3^3^	Cohort 1^4^
Number of subjects:				
Number of subjects planned, N:	8	8	8	12
Number of subjects randomized, N:	11	8	8	12
Number of subjects completed as planned, n (%):	8 (73)	7 (88)	8 (100)	12 (100)
Number of subjects withdrawn (any reason), n (%):	3 (27)	1 (13)	0	0
Withdrawn due to adverse events, n (%)	1 (9)	1 (13)	0	0
Withdrawn due to other reasons, n (%)	2 (18)	0	0	0
Demographics				
Age in years (mean (SD))	29.0 (8.66)	32.6 (10.03)	35.0 (7.62)	30.4 (8.34)
Sex (n (%))				
Female	0	0	0	0
Male	11 (100)	8 (100)	8 (100)	12 (100)
BMI (kg/m^2^) (mean (SD))	24.08 (1.585)	23.03 (0.829)	24.54 (2.894)	25.70 (2.884)
Height (cm) (mean (SD))	178.6 (6.52)	176.1 (5.06)	183.1 (7.68)	178.8 (7.63)
Weight (kg) (mean (SD))	77.05 (8.938)	71.41 (4.105)	82.50 (12.143)	82.33 (11.235)
Ethnicity (n (%))				
Hispanic or Latino	1 (9)	0	2 (25)	3 (25)
Not Hispanic or Latino	10 (91)	8 (100)	6 (75)	9 (75)
Race (n (%))				
African American/African Heritage	0	0	0	1 (8)
Asian – South East Asian Heritage	0	1 (13)	0	1 (8)
White – White/Caucasian/European Heritage	10 (91)	7 (88)	6 (75)	10 (84)
White – mixed race	1 (9)	0	0	0
Mixed race	0	0	2 (25)	0

^1^0.5 mg fasted, 2 mg fasted, 8 mg fasted, 40 mg fasted, placebo; ^2^8 mg fed, 60 mg fed, 125 mg fed, 125 mg fasted, placebo; ^3^250 mg fasted, 325 mg fed, 600 mg fed, 750 mg fed, placebo; ^4^simvastatin, simvastatin + 80 mg, simvastatin + placebo.

**Table 2.** Summary of on-treatment adverse events (≥ 2 subjects) by treatment, study 1.

	Placebo N = 24	GSK2647544												Total N = 27
		0.5 mg fasted N = 6	2 mg fasted N = 5	8 mg fasted N = 6	40 mg fasted N = 6	8 mg fed N = 6	60 mg fed N = 6	125 mg fed N = 6	125 mg fasted N = 5	250 mg fasted N = 6	325 mg fed N = 6	600 mg fed N = 6	750 mg fed N = 6	
Any event, n (%)	14 (58)	3 (50)	1 (20)	0	1 (17)	4 (67)	3 (50)	3 (50)	2 (40)	4 (67)	5 (83)	2 (33)	2 (33)	22 (81)
Headache, n (%)	6 (25)	2 (33)	0	0	1 (17)	3 (50)	1 (17)	1 (17)	1 (20)	1 (17)	2 (33)	0	1 (17)	11 (41)
Dermatitis contact, n (%)	4 (17)	0	0	0	0	0	2 (33)	0	1 (20)	1 (17)	0	0	1 (17)	9 (33)
Presyncope, n (%)	1 (4)	0	1 (20)	0	0	0	0	0	0	2 (33)	0	0	0	4 (15)
Myalgia, n (%)	1 (4)	0	0	0	0	0	0	1 (17)	0	1 (17)	0	1 (17)	0	4 (15)
Ventricular tachycardia, n (%)	0	1 (17)	0	0	0	1 (17)	0	1 (17)	0	0	0	0	0	3 (11)
Dizziness, n (%)	0	1 (17)	0	0	0	0	0	0	0	0	1 (17)	0	0	2 (7)
Fatigue, n (%)	2 (8)	0	0	0	0	0	0	0	0	0	0	0	0	2 (7)
Nasal congestion, n (%)	0	0	0	0	0	0	1 (17)	0	0	0	1 (17)	0	0	2 (7)
Nasopharyngitis, n (%)	0	0	0	0	0	0	0	0	0	0	1 (17)	1 (17)	0	2 (7)

**Table 3.** Summary of on-treatment adverse events (≥ 1 subjects) by treatment, study 2.

	Simvastatin	GSK2647544 + simvastatin	Placebo + simvastatin
	N = 12	N = 9	N = 3
	n (%)	n (%)	n (%)
Any AE	1 (8%)	6 (67%)	3 (100%)
Headache	0	2 (22%)	1 (33%)
Abdominal discomfort	0	3 (33%)	0
Application site rash	1 (8%)	0	2 (67%)
Abdominal distention	0	1 (11%)	0
Diarrhea		1 (11%)	0
Eructation	0	1 (11%)	0
Arthragia	0	1 (11%)	0
Back pain	0	1 (11%)	0
Pain in extremity	0	1 (11%)	0
Dizziness	0	0	1 (33%)
Nasopharyngitis	0	0	1 (33%)
Pollakiuria	1 (8%)	0	0

**Table 4.** Summary of GSK2647544 PK parameters following a single dose.

Parameter	Dose (mg)	N^1^	n^2^	Geometric mean (CVb%)	95% CI
GSK2647544, fasted state					
C_max_ (ng/mL)	0.5	6	6	2.69 (52.4)	1.60, 4.51
	2	5	5	5.30 (38.1)	3.36, 8.37
	8	6	6	14.6 (43.0)	9.51, 22.6
	40	6	6	77.3 (53.9)	45.5, 131
	125	5	5	208 (46.1)	121, 358
	250	6	6	370 (62.4)	202, 675
AUC_(0–_∞_)_ (ng×h/mL)	0.5	6	4	10.3 (19.9)	7.54, 14.1
	2	5	5	35.4 (45.8)	20.6, 60.8
	8	6	4	115 (70.7)	41.8, 317
	40	6	6	749 (20.2)	607, 924
	125	5	5	1,862 (22.4)	1,415, 2,451
	250	6	6	3,695 (48.8)	2,275, 6,001
AUC_(0-t)_ (ng×h/mL)	0.5	6	6	6.57 (49.4)	4.03, 10.7
	2	5	5	31.6 (47.3)	18.1, 55.2
	8	6	6	104 (59.6)	58.5, 186
	40	6	6	727 (19.0)	597, 886
	125	5	5	1843 (22.4)	1,399, 2,426
	250	6	6	3,650 (48.8)	2,247, 5,928
t_1/2_ (h)	0.5	6	4	2.38 (23.9)	1.63, 3.46
	2	5	5	4.22 (56.3)	2.20, 8.10
	8	6	4	7.83 (13.5)	6.32, 9.70
	40	6	6	12.3 (22.4)	9.73, 15.5
	125	5	5	10.7 (34.4)	7.08, 16.2
	250	6	6	11.5 (26.5)	8.74, 15.1
t_max_ (h)^3^	0.5	6	6	1.00 (1.0, 4.0)	NA
	2	5	5	2.00 (1.0, 4.0)	NA
	8	6	6	2.00 (0.5, 4.0)	NA
	40	6	6	4.00 (1.0, 4.0)	NA
	125	5	5	2.00 (1.0, 4.0)	NA
	250	6	6	3.00 (2.0, 6.0)	NA
GSK2647544, fed state					
C_max_ (ng/mL)	8	6	6	24.8 (20.9)	19.9, 30.8
	60	6	6	179 (30.5)	131, 245
	125	6	6	457 (28.8)	340, 614
	325	6	6	736 (46.6)	463, 1,172
	600	6	6	1,325 (36.0)	919, 1,912
	750	6	6	1,418 (21.9)	1,129, 1,780
AUC_(0–_∞_)_ (ng×h/mL)	8	6	6	165 (24.0)	129, 211
	60	6	5	1,207 (22.0)	921, 1,581
	125	6	6	2,506 (25.7)	1,922, 3,266
	325	6	6	5,198 (49.9)	3,168, 8,530
	600	6	6	9,595 (35.9)	6,656, 13,831
	750	6	6	11,375 (26.9)	8,620, 15,010
AUC_(0–t)_ (ng×h/mL)	8	6	6	152 (27.4)	115, 202
	60	6	6	1,103 (25.2)	850, 1,432
	125	6	6	2,480 (25.9)	1,897, 3,241
	325	6	6	5,137 (50.2)	3,124, 8,448
	600	6	6	9,495 (35.5)	6,612, 13,635
	750	6	6	11,199 (27.3)	8,450, 14,844
t_1/2_ (h)	8	6	6	9.40 (15.6)	7.99, 11.1
	60	6	5	11.2 (27.6)	7.98, 15.6
	125	6	6	11.0 (14.7)	9.39, 12.8
	325	6	6	12.2 (29.5)	9.04, 16.6
	600	6	6	12.3 (21.9)	9.80, 15.4
	750	6	6	13.3 (26.0)	10.2, 17.4
t_max_ (h)^3^	8	6	6	2.00 (1.0, 4.0)	–
	60	6	6	2.00 (1.0, 4.0)	–
	125	6	6	2.00 (2.0, 4.0)	–
	325	6	6	2.00 (1.0, 4.0)	–
	600	6	6	2.00 (1.0, 4.0)	–
	750	6	6	3.00 (2.0, 6.0)	–

^1^Number of subjects randomized; ^2^number of subjects with derived parameter; ^3^median (range).

**Table 5.** Summary of GSK2647544 PK parameters following a single dose (day 1) and repeat doses (day 7).

Parameter	Treatment^1^	N	n	Geometric mean (CVb%)	95% CI
AUC_(0–12)_ (ng×h/mL)	A1	9	9	1,064 (24.3)	(885, 1278)
	A1+S	9	9	2,272 (37.1)	(1,723, 2,994)
AUC_(0–∞)_ (ng×h/mL)	A1+S	9	9	4,041 (44.9)	(2,907, 5,617)
C_max_ (ng/mL)	A1	9	9	205 (31.5)	(162, 260)
	A1+S	9	9	370 (26.6)	(302, 452)
t_1/2_ (h)	A1+S	9	9	15.9 (49.5)	(11.1, 22.7)
t_max_ (h)^2^	A1	9	9	2.02 (1.98-3.97)	-
	A1+S	9	9	3.92 (2.00-3.98)	-

^1^A1 = GSK2647544 80 mg on day 1; A1+S = GSK2647544 80 mg b.i.d. for 7 days; ^2^median (range).

**Table 6.** Summary of simvastatin and simvastatin acid PK parameters, and comparison with and without co-administration of GSK2647544.

Analyte	Parameter	Treatment^1^	N	n	Geometric mean (CVb%)	95% CI	Ratio of geometric means^2^ (90% CI)
Simvastatin	C_max_ (ng/mL)	S1	12	12	1.46 (61.2)	(1.02, 2.09)	3.56 (2.53, 5.00)
		A1+S	9	9	4.60 (57.7)	(3.04, 6.94)	
		P1+S	3	3	2.01 (15.2)	(1.38, 2.93)	
	t_max_ (h)^3^	S	12	12	2.02 (1.00 – 4.03)	–	NA^4^
		A1+S	9	9	2.02 (2.00 – 3.98)	–	
		P1+S	3	3	2.00 (1.00 – 5.92)	–	
	AUC_(0–_∞_)_ (ng×h/mL)	S	12	11	5.34 (38.3)	(4.17, 6.85)	3.68 (2.53, 5.34)
		A1+S	9	9	17.6 (55.3)	(11.8, 26.2)	
		P1+S	3	1	9.97 (NA^4^)	NA^4^	
	AUC_(0-t)_ (ng×h/mL)	S	12	12	4.38 (60.1)	(3.08, 6.24)	4.25 (3.03, 5.97)
		A1+S	9	9	16.5 (55.8)	(11.0, 24.6)	
		P1+S	3	3	10.1 (11.4)	(7.58, 13.4)	
Simvastatin acid	C_max_ (ng/mL)	S	12	12	0.504 (79.3)	(0.324, 0.786)	2.30 (1.46, 3.63)
		A1+S	9	9	1.30 (51.5)	(0.898, 1.89)	
		P1+S	3	3	0.351 (40.6)	(0.133, 0.927)	
	t_max_ (h)^3^	S	12	12	4.04 (0.50 – 6.00)	–	NA^4^
		A1+S	9	9	5.92 (3.92 – 5.93)	–	
		P1+S	3	3	5.92 (4.00 – 11.95)	–	
	AUC_(0–_∞_)_ (ng×h/mL)	S	12	6	5.37 (42.8)	(3.50, 8.26)	1.50 (0.48, 4.71)
		A1+S	9	1	9.34 (NA^4^)	NA^4^	
		P1+S	9	0	ND^5^	ND^5^	
	AUC_(0-t)_ (ng×h/mL)	S	12	12	2.49 (290)	(0.963, 6.45)	3.09 (1.05, 9.03)
		A1+S	9	9	7.93 (44.7)	(5.72, 11.0)	
		P1+S	3	3	2.14 (62.8)	(0.510, 8.95)	

^1^S = simvastatin 10 mg SD; A1+S = simvastatin 10 mg SD plus GSK2647544 80 mg b.i.d. for 7 days; P1+S = simvastatin 10 mg SD plus GSK2647544 matched placebo b.i.d. for 7 days; ^2^comparison of parameters with and without co-administration of GSK2647544 for the 9 subjects who received simvastatin with GSK26476544 (A1+S) and simvastatin alone (S); ^3^median (range); ^4^not applicable; ^4^not determined.

**Table 7.** Summary of GSK2647544 PK model parameters estimates.

Parameter	Estimate	Inter-subject variability
Ka (1/h), absorption rate constant	0.587	0.139
CL/F (L/h), apparent central clearance	48.3	0.0273
V1/F (L), apparent central volume of distribution	181	0.0767
Q/F (L/h), apparent inter-compartment clearance	32.7	NE^1^
V2/F (L), apparent peripheral volume of distribution	331	NE^1^

^1^Not estimated.
